# Roxadustat for renal anemia: current clinical experience

**DOI:** 10.3389/fphar.2025.1712869

**Published:** 2026-01-07

**Authors:** Jialing Ji, Zhongtang Li, Shuping Fang, Zuolin Li

**Affiliations:** 1 Department of Pediatrics, The Fourth Affiliated Hospital of Nanjing Medical University, Nanjing, Jiangsu, China; 2 Department of Pediatrics, Zhongda Hospital, Southeast University School of Medicine, Nanjing, Jiangsu, China; 3 Institute of Nephrology, Nanjing Lishui District People’s Hospital, Lishui/Nanjing, China; 4 Institute of Nephrology, Zhongda Hospital, Southeast University School of Medicine, Nanjing, Jiangsu, China

**Keywords:** advantages, clinical experience, renal anemia, roxadustat, safety concerns

## Abstract

Anemia represents one of the most prevalent complications of chronic kidney disease (CKD). Investigation of the regulation of erythropoietin production led to the discovery of oxygen-sensing mechanisms, which are now being targeted therapeutically (hypoxia-inducible factor prolyl hydroxylase inhibitors, HIF-PHI). Roxadustat, a first-in-class of HIF-PHI, achieved its first approval in China in December 2018 for managing anemia in adult patients with CKD. Over the past 6 years, we have gained substantial real-world experience regarding its therapeutic application. Accordingly, accumulated evidence demonstrates that roxadustat is superior to placebo and non-inferior to current standard-of-care erythropoiesis-stimulating agents in correcting and maintaining hemoglobin concentration at target levels among patients with non-dialysis dependent CKD and incident and dialysis dependent CKD. However, given the pleiotropic effects of HIF activation, the pharmacologic activation of HIF in patients with anemia of CKD is also likely to have effects beyond erythropoiesis and iron metabolism. In this review, we synthesize clinical insights from these novel therapeutics and highlight some of the outstanding questions relevant to their clinical use. This study may provide theoretical framework and practical evidence for the clinical application of other HIF-PHIs.

## Introduction

Anemia, a condition associated with a high burden of morbidity and adverse clinical outcomes, represents one of the most prevalent complications in patients with chronic kidney disease (CKD) (Hanna et al., 2021). Although anemia management was revolutionized in the 1980s with the introduction of recombinant human erythropoietin (EPO), adverse effects including worsening hypertension, seizures, dialysis access clotting, adverse cardiovascular events, and even increased risk of death, were noted in patients receiving erythropoiesis-stimulating agents (ESAs). Recently, accumulating evidence demonstrated that dysregulated oxygen sensing in the diseased kidney is the underlying mechanism resulting in anemia (Li et al., 2022; Schödel and Ratcliffe, 2019). Understanding of the molecular mechanisms underlying anemia of CKD holds promise for developing new pharmacologic agents that more closely target the underlying mechanisms for improved efficacy and minimized treatment-related adverse outcomes.

Investigation of the regulation of EPO production, one of the classical responses to hypoxia, has revealed fundamental oxygen-sensing mechanisms that are currently being therapeutically targeted. Therefore, a new category of drugs was developed, hypoxia-inducible factor prolyl hydroxylase inhibitors (HIF-PHIs) (Gupta et al., 2017). Roxadustat (FG-4592), the first-in-class of HIF-PHI, regulates the balance between HIF synthesis and degradation rates by mimicking hypoxia, thereby correcting anemia. Following its initial approval in China for anemic adults with CKD in December 2018 (Dhillon, 2019), roxadustat has subsequently received regulatory authorization in 15 additional countries including Japan, Chile, South Korea, the European Union, Iceland, Mexico, Norway, Russia, South Africa, and United Kingdom. Over the past 6 years, we have gained substantial real-world experience regarding its therapeutic application. In this review, we synthesize clinical insights from these novel therapeutics and highlight some of the outstanding questions relevant to their clinical use.

## Oxygen-sensing mechanisms

Elucidation of the exact mechanism of gene expression (mainly *Epo*) by oxygen has established the foundational molecular framework for understanding human oxygen-sensing systems. The HIF, a heterodimer comprising an inducible α subunit (HIF-α) and a constitutively expressed β subunit (HIF-β), was identified as the key transcription factor regulating these transcriptional responses. Mechanistically, the oxygen-sensitive signal that controls HIF activity is generated by a series of regulatory enzymes that catalyse the hydroxylation of specific prolyl and asparaginyl residues in the HIF-α subunits. Subsequent investigations have systematically characterized the prolyl hydroxylase enzymes governing this regulatory modification. To date, three evolutionarily conserved genes (*EGLN1, EGLN2 and EGLN3*) encoding prolyl hydroxylase domain (PHD) enzymes (PHD2, PHD1 and PHD3) have been identified in humans. Given the characteristic of these PHD enzymes being regulated by oxygen, the oxygen-regulated signals that govern the activity of HIF are generated by enzymatic oxygen sensors (Semenza, 2012).

HIF, as a master transcription factor of oxygen homeostasis, orchestrates multiple cellular processes through transcriptional activation of target genes. On one hand, HIF directly stimulates transcription of the *Epo* gene in the kidneys and liver. On the other hand, HIF activation upregulated the expression of genes involved in iron transport, enhancing its uptake and absorption. Moreover, hepcidin inhibition is achieved predominantly through indirect pathways mechanisms, allowing mobilization of iron from stores. In addition, a series of other genes expression are stimulated that activate pathways involved in multiple biological processes (energy metabolism, angiogenesis, cell proliferation, immune responses, and extracellular matrix remodeling) (Luo et al., 2022). Collectively, a deep understanding of the oxygen-sensing mechanism provides a crucial targeted strategy for anemia management.

## HIF-PHIs: a novel way to treat renal anemia

**FIGURE 1:**
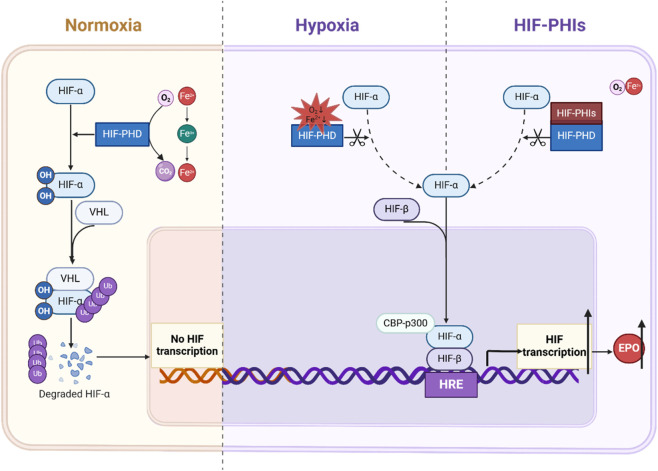
Mechanisms of HIF signaling regulation and pharmacological mechanism of HIF-PHI. Under normoxia, the prolyl residue of HIF-α was hydroxylated by PHD, so that it could be recognized by VHL and then binds to the E3 ubiquitin ligase, resulting in the degradation of HIF-α. Under hypoxia, the activity of PHD was inhibited, allowing HIF-α expressed stably so that it could be translocated to the nucleus, where it dimerized with HIF-β to became an activated complex, and then bind to the HRE on DNA, recruited the transcriptional coactivator CBP/p300, resulting in the activation of target gene transcription. Roxadustat is an oral, reversible, small molecular PHD inhibitor that mimics the natural response to hypoxia, resulting in transient transcription of a variety of genes to adapt to hypoxia, including EPO. CBP: CREB-binding protein; EPO; Erythropoietin; HIF: Hypoxia-inducible factor; HRE: Hypoxia response element; PHD: Prolyl hydroxylase; VHL: von Hippel-Lindau tumor suppressor; Ub: Ubiquitylation.

Pharmacological manipulation of the HIF pathway by the HIF-PHI has emerged as a novel approach for treating anemia in patients with CKD. Roxadustat, a first-in-class HIF-PHI used for treating anemia in CKD patients, achieved its first approval in China in December 2018. Accumulating evidence indicates that roxadustat stabilizes HIF and stimulates HIF-target gene expression, thereby correcting anemia systematically and physiologically (Figure 1).

Roxadustat has been clinically employed in renal anemia management for 6 years. Over the past 6 years, we have gained extensive clinical experience regarding its therapeutic application. Current clinical data demonstrates that roxadustat is superior to placebo and/or non-inferior to ESAs in correcting and/or maintaining hemoglobin (Hb) concentration at target levels among patients with non-dialysis dependent CKD (NDD-CKD) and incident and dialysis dependent CKD (DD-CKD). Notably, there are significant characteristic differences among the three populations, and direct clinical evidence comparing roxadustat treatment outcomes across these groups is currently lacking. Furthermore, given that response to roxadustat varies by inflammation burden, iron availability, and ESA resistance, we will separately discuss the therapeutic effects of roxadustat in these three populations.

### Roxadustat in the management of anemia in NDD-CKD

Available phase 2 and 3 trials of roxadustat in NDD-CKD are summarized in Table 1. In the *versus* placebo trials, roxadustat was superior in achieving and maintaining target Hb levels for up to 5 years. Notably, in the first phase 2 study conducted in China, Hb response in CKD patients with NDD-CKD was dose-dependent and significantly higher in the roxadustat arms than in the placebo arm (88.5% and 93.1% of the low- and high-dose cohorts, respectively, *versus* in 25.9% of placebo subjects; *P* < 0.0001) (Chen et al., 2017). Meanwhile, in the first phase 3 trial conducted in China (randomized, double-blind, and placebo-controlled trial), roxadustat was superior to placebo in increasing Hb levels in CKD patients with NDD-CKD. In this trial, the mean (±SD) change from baseline in the Hb level was an increase of 1.9 ± 1.2 g/dL in the roxadustat group and a decrease of 0.4 ± 0.8 g/dL in the placebo group (*P* < 0.001) (Chen et al., 2019a). Further, a pooled analysis of the ALPS (Shutov et al., 2021), ANDES (Coyne et al., 2020) and OLYMPUS (Fishbane et al., 2021) trials of roxadustat *versus* placebo in 4,277 patients showed a greater increase in Hb (1.9 g/dL vs. 0.2 g/dL), and a greater Hb response (80% vs. 9%) in the first 52 weeks of treatment in the roxadustat arm (Provenzano et al., 2021a).

**TABLE 1:** Phase 2 or 3 trials of roxadustat to treat anemia in non–dialysis-dependent CKD.

Clinical trial number	Phase	Location	Groups	Number of patients	Roxadustat dose	Study duration	Patients type
NCT00761657	2	United States	Placebo-controlled	88/28	1.5, 2.0 or 0.7 mg/kg; TIW	6 weeks	Stage 3 or 4 CKD
NCT01244763	2b	United States	-	145	Dosage titration based on the cohort-specific adjustment rules	24 weeks	NDD-CKD
NCT01599507	2	China	Placebo-controlled	61/30	Low (1.1–1.75 mg/kg) or high (1.50–2.25 mg/kg); TIW	8 weeks	CKD not receiving dialysis
NCT02652819	3	China	Placebo-controlled	101/51	70 mg (weighing 40 to <60 kg) or 100 (weighing ≥60 kg); TIW	26 weeks	CKD not receiving dialysis
NCT01964196	2	Japan	Placebo-controlled	80/27	(50, 70, or 100 mg); TIW	24 weeks	NDD-CKD
NCT01750190	3	United States	Placebo-controlled	606/306	70 mg (weighing 45 to <70 kg or 100 mg (weighing ≥70 kg); TIW	52 weeks	Stage 3–5 CKD and not on dialysis
NCT02988973	3	Japan	Darbepoetin alfa-controlled	131/71	Initial dose conversion to roxadustat, based on ESA use	24 weeks	NDD-CKD
NCT01887600	3	Russia	Placebo-controlled	391/203	70 mg (weighing 45 to <70 kg or 100 mg (weighing 70–160 kg); TIW	104 weeks	NDD-CKD
NCT02174627	3	United States	Placebo-controlled	1393/1388	The starting dose was 70 mg TIW. The maximum dose was capped at the lower of 3.0 mg/kg or 300 mg per dose administration	164 weeks	NDD-CKD
NCT02021318	3	United Kingdom	Darbepoetin alfa-controlled	323/293	70 mg (weighing 45 to <70 kg or 100 mg (weighing 70–160 kg); TIW	104 weeks	NDD-CKD

USA, united states of america; UK, united kingdom; ESA, erythropoiesis-stimulating agent; NDD-CKD: non-dialysis-dependent chronic kidney disease.

Moreover, in the *versus* ESAs trials, roxadustat is non-inferior to in correcting and/or maintaining Hb at target levels in NDD-CKD patients (Barratt et al., 2021a; Akizawa et al., 2021a). Finally, a systematic review and meta-analysis on efficacy of roxadustat for anemia in patients with NDD-CKD revealed that roxadustat significantly increased the Hb response rate compared with placebo in the NDD-CKD group (Liu et al., 2021; Tang et al., 2021).

### Roxadustat in the management of anemia in DD-CKD

Available phase 2 and 3 trials of roxadustat *versus* ESAs in DD-CKD are summarized in Table 2. In the anemic patients with DD-CKD, 59.1%, 88.9% (*P* = 0.008) and 100% (*P* = 0.0003) of the low-, medium- and high-dose subjects maintained their Hb levels *versus* 50% of the epoetin alfa-treated subjects (Chen et al., 2017). Similarly, the phase 3 trials demonstrated that roxadustat was non-inferior to ESAs in Hb correction and maintenance in studies of incident (Provenzano et al., 2021b; Fishbane et al., 2022; Charytan et al., 2021) or prevalent (Fishbane et al., 2022; Charytan et al., 2021; Csiky et al., 2021; Chen et al., 2019b) DD-CKD patients. Accordingly, the first randomized, open-label, active-controlled (epoetin alfa), phase 3 trial conducted in China, evaluating the noninferiority of roxadustat found that roxadustat led to a numerically greater mean (±SD) change in Hb level from baseline to weeks 23 through 27 (0.7 ± 1.1 g/dL) than epoetin alfa (0.5 ± 1.0 g/dL) and was statistically noninferior (95% confidence interval [CI], −0.02–0.5) (Chen et al., 2019b).

**TABLE 2:** Phase 2 or 3 trials of roxadustat to treat anemia in dialysis-dependent CKD.

Clinical trial number	Phase	Location	Groups	Number of patients	Roxadustat dose	Study duration	Patients type
NCT01147666	2	United States	Roxadustat VS. Epoetin alfa	41/13	1.0, 1.5, 1.8, or 2.0 mg/kg; TIW	6 weeks	HD
NCT01147666	2	United States	Roxadustat VS. Epoetin alfa	67/23	1.0, 1.5, 1.8, or 2.0 mg/kg; TIW	19 weeks	HD
NCT01596855	2	China	Roxadustat VS. Epoetin alfa	74/22	1.1-1.8, 1.5-2.3 or 1.7–2.3 mg/kg; TIW	6 weeks	HD
NCT02652806	3	China	Roxadustat VS. Epoetin alfa	204/100	100 mg (weight 45 to 60 Kg), 120 mg (weight >60 Kg); TIW	27 weeks	HD + PD
NCT02952092	3	Japan	Roxadustat VS. Darbepoetin alfa	150/151	70 mg or 100 mg	24 weeks	HD
NCT02780726	3	Japan	Roxadustat	56	Randomized to 50 or 70 mg; TIW	24 weeks	PD
NCT02273726	3	United States	Roxadustat VS. Epoetin alfa	370/371	70, 100, 150, or 200 mg TIW based on the patient’s prescribed pre-study ESA dose	52 weeks	HD + PD
EudraCTnumber 2013-001497-16	3	Europe and United States	Roxadustat VS. Epoetin alfa or darbepoetin alfa	414/420	20, 50, 100 mg TIW based on the patient’s prescribed pre-study ESA dose	104 weeks	HD + PD
NCT02052310	3	United States, Europe, South America, and Asia	Roxadustat VS. Epoetin alfa	522/521	70 mg (weight ≤70 Kg), 100 mg (weight >70–160 Kg); TIW	52 weeks	HD + PD
ChiCTR2000035054	3	China	Roxadustat VS. Epoetin alfa	86/43	100 mg (weighing 45 to <60 kg), 120 mg (weighing ≥60 kg); TIW	24 weeks	PD
NCT02174731	3	United States, Europe, Asia, Australia, Latin America	Roxadustat VS. Epoetin alfa	1051/1055	70–200 mg TIW, based on prior ESA dose	52 weeks	HD + PD

United States, united states of america; ESA, erythropoiesis-stimulating agent; HD, hemodialysis; PD, peritoneal dialysis.

Further, a pooled analysis of the HIMALAYAS (Provenzano et al., 2021b), ROCKIES (Fishbane et al., 2022), SIERRAS (Charytan et al., 2021), and PYRENEES (Csiky et al., 2021) trials of roxadustat *versus* ESAs in 4,714 patients showed that Hb changes from baseline to weeks 28–36 achieved non-inferiority for roxadustat (Barratt et al., 2021b). Finally, recently, a systematic review and meta-analysis including 10 different RCTs and 5,698 DD-CKD patients revealed that compared to the ESAs group, the roxadustat group showed increased Hb levels (95%CI, 0.14 to 0.36; *P* < 0.00001).

Moreover, the efficacy of roxadustat for peritoneal dialysis patients with renal anemia was explored in China. As expected, in a randomized controlled trial, cumulative response rate was 96% in the roxadustat group and 92% in the ESAs group at week 24, confirming its effectiveness in correcting and maintaining target Hb levels in this population (Hou et al., 2022a). Additionally, the Hb level and its change in the peritoneal dialysis group was significantly higher compared to that in the hemodialysis group despite the higher dose of roxadustat in the latter group (Zhang et al., 2024). More interestingly, a randomized study on the evaluation of dose response suggests that a lower starting dose of roxadustat effectively achieves the Hb target as standard-dose does among Chinese peritoneal dialysis patients (Yang et al., 2021). Notably, although the pharmacokinetic profile of roxadustat of peritoneal dialysis patients were comparable to those of dialysis patients was not influenced by the type of dialysis (Takada et al., 2022), the median *half-life (t*_*1/2*_ *)* tended to be shorter in subjects with severely impaired kidney function than in those with hemodialysis (Groenendaal-van de Meent et al., 2021). Given the critical role of residual renal function, this may explain why peritoneal dialysis patients exhibit a favorable response to roxadustat treatment.

### Patients with anemia of inflammation

In clinical practice, management of anemia of inflammation is often challenging. Interestingly, roxadustat appears to open a new door for the treatment of renal anemia in these patients. In a randomized, open-label, active-controlled, phase 3 trial conducted in China in patients undergoing dialysis, mean Hb levels were comparable between those with elevated C-reactive protein (CRP) level and those with normal CRP level (11.2 ± 0.9 g/dL vs. 11.3 ± 1.0 g/dL) among roxadustat-treated patients (Chen et al., 2019b). Similar findings were reported in clinical research conducted on NDD-CKD patients (Fishbane et al., 2021).

Additionally, recently, our group conducted a retrospective cohort study in China to evaluate the efficacy of roxadustat on renal anemia with systemic microinflammation. The results revealed that roxadustat could significantly increase Hb level in patients with macroinflammation (hsCRP ≥ 10 mg/L). Notably, anemia improvement is observed in patients with high CRP levels at the same dose as in the non-inflammatory state (Tu et al., 2024). Further, a systematic review evaluating the impact of CRP on the effect of roxadustat for the treatment of anemia in CKD revealed no significant difference in Hb change from baseline between patients with CRP ≥ ULN and CRP < ULN. More importantly, an increase in ESA dose over time was observed in the CRP ≥ ULN group, while the dose of roxadustat remains constant over time and is not influenced by the baseline levels of CRP (Luo et al., 2024). Therefore, roxadustat are efficacious in correcting and maintaining Hb levels in patients with anemia associated with inflammation.

### Other specific populations

With the emergence of roxadustat came the hope that using the oral drug manages those difficult-to-manage patients with anemia of CKD, particularly in ESA-hyporesponsive patients. Recently, the results of *post hoc* analysis of two double-blind, randomized, phase 3 study in DD-CKD or in NDD-CKD patients assessed the impact of factors associated with ESA hyporesponsiveness on roxadustat and darbepoetin alfa doses indicated that roxadustat doses required to maintain target Hb appear to be less affected by factors that underlie ESA hyporesponsiveness, relative to ESA (Akizawa et al., 2021b; Akizawa et al., 2021c). Additionally, in almost all clinical trial, roxadustat treatment was observed to reduce serum hepcidin levels in patients with NDD-CKD and DD-CKD, indicating that roxadustat may help to ameliorate ESA hyporesponsiveness induced by functional iron deficiency. Indeed, in a prospective study conducted in China included 30 dialysis patients with ESA-hyporesponsive anemia, the responsive rate was 93.3% (Song et al., 2023). Although roxadustat may be beneficial for patients hyporesponsive to ESAs, further studies are needed to demonstrate the efficacy, dose requirements, and safety of roxadustat in patients with ESA hyporesponsiveness.

## Real-world evidence of roxadustat treatment

The core merit of real-world study is that it closely reflects real-world clinical practice. Recently, a real-world study analyzed 6,414 hemodialysis patients with renal anemia conducted by Zhang et al. indicated that roxadustat showed significantly greater Hb increases *versus* ESA at 6–12 months (least-squares mean difference: 0.46 g/dL, p = 0.04) and Hb response rates were higher with roxadustat (84.0% vs. 76%, p < 0.01) (Zhang et al., 2025). Meanwhile, another real-world study evaluating the long-term effectiveness and safety of roxadustat among patients with CKD-anemia with and without dialysis in China also reported consistent findings with acceptable safety (Du et al., 2025), supporting its use in real-world settings. Notably, there is a paucity of real-world data from non-Asian countries, and the efficacy and safety in populations from other countries warrant further exploration.

## Treatment initiation and monitoring

To date, no clinical trials have been performed to establish new thresholds/targets for the initiation of HIF-PHI therapy, but it is reasonable to use the same Hb thresholds as those recommended or suggested for ESA therapy. Although previous studies have recommended starting dose of roxadustat treatment, it is generally recommended that roxadustat is prescribed at the lowest dose needed to improve symptoms attributable to anemia and to avoid red blood cell transfusions (Kidney Disease: Improving Global Outcomes KDIGO Anemia Work Group, 2025). This point considers the possibility that, based on mechanism of action, higher roxadustat doses may result in adverse events.

Because maintaining higher Hb levels and a rapid increase in the Hb levels are risk factors for poor outcomes, clinicians should be aware of Hb overshoot caused by roxadustat in clinical practice. Recently, a retrospective study to examine whether early overshoot frequently occurs after switching from ESA to roxadustat was conducted. Interestingly, it was found that 34.8% in the roxadustat group and 3.2% in the ESA group had Hb overshoot within the 8-week visit. Among the patients with Hb overshoot in the roxadustat group, the Hb levels were maintained close to baseline 4 weeks after roxadustat discontinuation (Tamaki et al., 2025). These data suggested that more frequent monitoring of Hb levels is mandatory. Moreover, a younger age and higher baseline Hb and hematocrit levels were risk factors for Hb overshoot. Thus, clinicians should be aware of Hb overshoot and emphasize the importance of early Hb level monitoring. To date, the ideal frequency of monitoring is uncertain. It is generally recommended that the Hb level should be monitored 2–4 weeks after initiation or dose changes and subsequently, every 4 weeks during roxadustat therapy (Kidney Disease: Improving Global Outcomes KDIGO Anemia Work Group, 2025).

Of note, although some clinical studies indicated that the combination of roxadustat and rHuEPO may have a better effect on improving anemia symptoms in hemodialysis patients (Wei et al., 2023; Huang et al., 2025), ESA coupled with different doses of Roxadustat for treatment of renal anemia in patients with CKD, including those with ESA hyporesponsiveness, was not recommended or suggested (Kidney Disease: Improving Global Outcomes KDIGO Anemia Work Group, 2025).

## Optimal Hb targets for the correction of anemia

Current Hb targets for the correction of anemia mainly are based on clinical trials (ESAs) conducted several years ago or clinical practice guidelines for diagnosis and treatment of renal anemia in China (Ku et al., 2023; Work Group on guidelines for, 2021) or the KDIGO Clinical Practice Guideline for Anemia (KDIGO Anemia Work Group, 2012). Because phase 2 and 3 trials of roxadustat were designed primarily for efficacy and safety evaluation and to meet criteria of guideline-recommended Hb targets, no roxadustat trials to date have compared higher Hb targets with the currently recommended lower Hb targets for CKD patients. Overall, the available data do not provide a rationale for targeting higher Hb levels with roxadustat than the currently recommended targets established using ESAs.

## Implications for iron management during the correction of anemia

Iron supplementation is also a critical cornerstone of anemia management. Although data from clinical trials suggest that Roxadustat enhanced iron availability while requiring less intravenous iron use (Wu et al., 2024; Pergola et al., 2022), there was consensus that iron metabolism status needs to be assessed before initiating treatment with roxadustat (Geng et al., 2024) and roxadustat therapy will not eliminate the need for iron replacement in CKD patients.

Previously, roxadustat has been shown to reduce hepcidin and ferritin levels and increase transferrin and total iron-binding capacity (Pergola et al., 2022; Hou et al., 2022b). Furthermore, a randomized, phase 4 study recently indicated that compared to the rHuEPO, roxadustat treatment was associated with decreased hepcidin, and increased transferrin, soluble transferrin receptor, and total iron-binding capacity, although no significant difference between roxadustat and rHuEPO in iron absorption was found (Ganz et al., 2023). These findings suggest that roxadustat may stimulate the release of iron from storage and increase its utilization. It is generally recommended that patients with NDD-CKD and patients on peritoneal dialysis with serum ferritin <100 μg/L and/or transferrin saturation (TSAT) < 20% and hemodialysis patients with serum ferritin <200 μg/L and/or TSAT<20% should be treated with iron. Oral iron supplementation may be preferred, and the results of randomized open-label studies in patients receiving hemodialysis and peritoneal dialysis have shown that oral iron has the same Hb-increasing efficacy as intravenous use (Besarab et al., 2016). Intravenous iron supplementation may be considered when oral iron is not tolerated.

Currently, the timing of iron treatment, the timing and frequency of monitoring of parameters of iron metabolism, and appropriate iron treatment target have not been clarified during the period of anemia correction and maintaining with roxadustat. Based on clinical experience, regular monitoring of parameters of iron metabolism should be performed at least monthly for patients in the initial treatment stage, and at least every 3 months for patients in the maintenance treatment stage of anemia or those with stable Hb.

Additionally, both the European Medicines Agency (Stoumpos et al., 2024) and the Asian Pacific Society of Nephrology (Yap et al., 2021) emphasize that iron status should be evaluated before HIF-PHI are used. Correcting iron deficiency before initiation of HIF-PHI (ferritin >100 ng/mL and TSAT >20%) for all CKD patients were strongly suggested. Iron deficiency should be avoided because it is associated with thromboembolic events, lower physical health-related quality of life (Guedes et al., 2021a), higher rates of cardiovascular events, and higher mortality (Guedes et al., 2021b).

## Advantages of roxadustat therapy

Roxadustat is an oral, potent, reversible, small molecular PHD inhibitor that mimics the natural response to hypoxia, resulting in transient transcription of a variety of genes to adapt to hypoxia, including EPO, EPO receptor, and iron metabolism. The clinical practice has demonstrated the efficacy of roxadustat in improvement of renal anemia. More interestingly, roxadustat increases and maintains Hb level independent of the baseline inflammation and iron state, which may be particularly significance for difficult-to-treat patients with anemia of CKD. Therefore, roxadustat offers distinct advantages over conventional ESAs in anemia correction.

Firstly, one of the most striking superiorities of roxadustat, in addition to its oral route of administration, is the ability to stimulate endogenous EPO gene expression, as substantially increasing serum EPO level within or near a physiological level (Provenzano et al., 2020). This avoids the potential adverse effects associated with supraphysiological ESA dosing, such as increased cardiovascular risk and mortality observed in ESA-treated patients.

**FIGURE 2:**
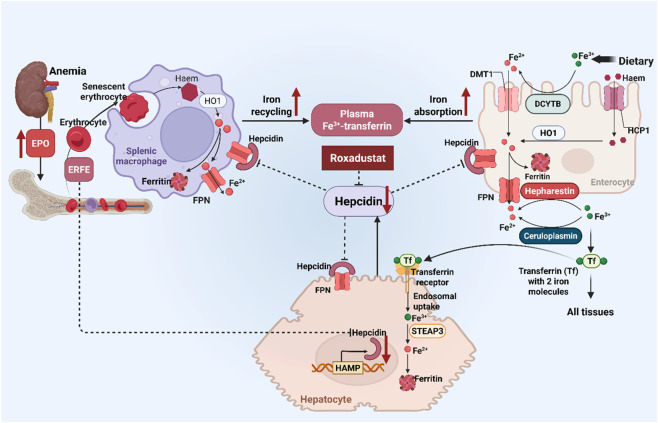
Roxadustat improves iron metabolism. The effects of roxadustat on iron metabolism is one of the most striking superiorities for anemia management. The hepcidin-FPN axis is the principal regulator of extracellular iron homeostasis in health and disease. Accordingly, hepcidin is internalized with FPN and both are degraded in lysosomes, which inhibits the release of iron from enterocytes of the duodenum and from macrophages. Roxadustat suppresses hepcidin expression, which increases FPN expression on hepatocytes, enterocyte, and macrophages, resulting in increased iron mobilization from internal stores and iron absorption from the intestinal tract. Collectively, roxadustat improves iron metabolism as well as stimulating EPO production to promote erythrocytes production. DCYTB: Duodenal cytochrome B; DMT1: Divalent metal transporter 1; EPO: Erythropoietin; FPN: Ferroportin; Tf: Transferrin; HO-1, heme oxygenase-1; HCP1, Haem carrier protein 1.

Secondly, roxadustat is confirmed to correct renal anemia through improving iron metabolism (Figure 2). It enhances enteric iron absorption by stimulating the expression of target genes, such as divalent metal transporter-1 and duodenal cytochrome B (Haase, 2011). Concurrently, roxadustat-mediated HIF pathway regulates the transcription of transferrin, transferrin receptor, heme oxygenase-1, and ferroportin, all of which are essential for improvement of disordered iron metabolism and erythropoietic activity. Moreover, the hepcidin-lowering effect of roxadustat promotes erythropoiesis in erythroblasts, since reduced hepcidin allows mobilization of iron from stores, enhance iron utilization (Li et al., 2020). Together, roxadustat may be more efficacious in correcting anemia despite chronic inflammation, where anemia management is often challenging in clinical practice.

Thirdly, due to its mechanism of action, roxadustat therapy represents a potential therapeutic alternative for the management of anemia in difficult-to-manage patients with CKD. Growing evidence suggests that roxadustat may theoretically be more effective in treating patients who are hyporesponsive to ESAs because of chronic inflammation or functional iron deficiency. Indeed, roxadustat may make erythropoiesis more “sensitive” through various potential mechanisms (Raichoudhury and Spinowitz, 2021).

Finally, roxadustat is a kind of orally bioavailable, small molecular inhibitor that inhibits PHD reversibly and transiently. With a short half-life, roxadustat, usually administered once or thrice weekly, enables HIF transcriptional activity to return to baseline between doses, which results in stimulation of EPO production in a titratable manner. Moreover, compared with currently available ESAs, roxadustat are orally administrative with no risk of developing anti-EPO antibodies that cross-react with EPO. Additionally, the result from a prospective study to determine red blood cell survival in Chinese long-term hemodialysis patients treated with Roxadustat found that roxadustat treatment seems to significantly increase the lifespan of red blood cell (Zhao et al., 2023). Furthermore, roxadustat have additional effects of cholesterol-lowering, as evidenced by multiple phase 2 and 3 clinical trials (Zhou et al., 2023; Lei et al., 2022). Mechanistically, it was found that HIF-1 accelerates degradation of HMG-CoA reductase in the liver through activation of insulin-induced gene two transcription, leading to reduced cholesterol synthesis (Hwang et al., 2017). In addition, HIF-1 also stimulates lipin one gene expression, which contributes to triglyceride accumulation in cells (Mylonis et al., 2012).

Taken together, roxadustat represents a superior anemia treatment option for CKD patients, particularly those with ESA hyporesponsiveness, iron intolerance, or chronic inflammation. Its unique mechanism addresses ESA limitations while offering oral convenience, improved safety profiles, and efficacy in clinically challenging scenarios.

## Safety concerns of roxadustat therapy

Roxadustat, as a pan-PHD inhibitor, induces activity of both HIF-1 and HIF-2, which may potentially lead to on-target and off-target adverse effects. Recently, some scholars have proposed the novel concept that roxadustat was more than an erythropoietic agent (Angeletti and Cravedi, 2024). To date, although roxadustat is generally well-tolerated, several critical safety concerns associated with roxadustat treatment remain to be addressed.

### Cardiovascular outcomes

Although the underlying mechanisms remain poorly understood, cardiovascular outcomes associated with roxadustat in anemia patients with CKD remains a critical concern. Given the real-word condition in clinical practice, the non-inferiority or potential superiority of roxadustat regarding major adverse cardiac events (MACEs) risks is primarily evaluated based on recommendations from ESAs.

#### NDD-CKD populations

In three phase 3, double-blind studies of roxadustat *versus* placebo evaluating the cardiovascular safety of renal anemia in NDD-CKD (n = 4,277), no increased risks were observed for MACE (HR 1.10; 95% CI, 0.96–1.27), MACE+ (HR 1.07; 95% CI, 0.94–1.21), and all-cause mortality (HR 1.08; 95% CI, 0.93–1.26) in patients treated with roxadustat *versus* those treated with placebo (Provenzano et al., 2021a). Meanwhile, in four phase 3, randomized, open-label studies of roxadustat *versus* ESA in patients with NDD-CKD or incident-to-dialysis CKD (n = 2,142), no evidence of increased risk was observed for MACE (HR 0.79; 95% CI, 0.61–1.02), MACE+ (HR 0.78; 95% CI, 0.62–0.98), and all-cause mortality (HR 0.78; 95% CI, 0.57–1.05) with roxadustat compared with ESA in patients with anemia who have NDD-CKD or incident-to-dialysis CKD (Barratt et al., 2023). However, the pooled analyses for roxadustat did not have prespecified non-inferiority margins that were agreed upon by the Food and Drug Administration (FDA), despite the absence of evidence for increased cardiovascular outcomes or all-cause mortality risks. Additionally, a separate roxadustat trial reported a higher mortality risk compared with placebo (18.9% vs. 15.5%) (Fishbane et al., 2021), which led to the FDA not approving roxadustat for marketing in the United States. However, to date, no subsequent studies explored the potential impact of roxadustat treatment on mortality in anemic patients.

Notably, despite the adverse outcomes, roxadustat exhibited favorable tolerability in Chinese patients. Accordingly, a phase 3 trial carried out in the Chinese populations found that there were no increased risks of cardiac disorder and all-cause mortality in NDD-CKD patients treated with roxadustat *versus* those treated with placebo (Chen et al., 2019a). Consequently, roxadustat is considered non-inferior to placebo or conventional ESAs for cardiovascular outcomes in Chinese NDD-CKD patients.

#### DD-CKD populations

A consensus has emerged from clinical studies (including Chinese cohorts) that roxadustat generally met non-inferiority criteria for MACE in cardiovascular outcome trials involving DD-CKD populations. In the four phase 3, randomized, open-label studies comparing roxadustat to ESAs in the DD-CKD population (n = 4,714), it was reported that roxadustat was non-inferior to ESA for MACE (HR 1.09; 95% CI, 0.95–1.26), MACE+ (HR 0.98; 95% CI, 0.86–1.11), and all-cause mortality (HR 1.13; 95% CI, 0.95–1.34) in the entire cohort and similar to the incident dialysis and stable dialysis subgroups (Barratt et al., 2021b). Furthermore, a recent meta-analysis (including 8 RCTs) indicated that roxadustat did not cause cardiovascular-related events in anemic patients with dialysis (RR 1.094; 95% CI, 0.925 to 1.293; *P* = 0.294). Additionally, no significant differences in cardiovascular event risk were observed when comparing roxadustat to placebo to placebo (RR 1.049; 95% CI, 0.918 to 1.200; *P* = 0.479) or ESA (RR 1.066; 95% CI, 0.919 to 1.235; *P* = 0.398) in CKD patients with anemia (Tian et al., 2024).

Despite meeting non-inferiority criteria for MACE in cardiovascular outcome trials involving DD-CKD populations, controversy has surrounded interpretation of the relevant data for roxadustat in this context. Currently, roxadustat have received marketing authorization in China, Japan, European Medicines Agency and other countries, while the FDA denied approval of roxadustat due to safety concerns.

### Thromboembolic events

Thrombosis-related events represent critical safety concerns, particularly in patients undergoing hemodialysis. The results of pooled analysis of pivotal, global studies of roxadustat in the NDD-CKD and the DD-CKD populations revealed that the incidence of vascular access thrombosis and deep vein thrombosis in patients treated with roxadustat was higher than that in the control group (Provenzano et al., 2021a; Barratt et al., 2021b). Meanwhile, the Asian Pacific Society of Nephrology does not recommend HIF-PHI for patients with any history of thrombotic events (Yap et al., 2021). However, the current data do not clearly indicate the risk differences among patients with different levels of thrombosis risk. Although a preclinical study reported that roxadustat does not affect platelet production, activation and thrombosis formation (Zhao et al., 2021), the effects of roxadustat on thrombosis-related events still need to be further studied. In clinical practice, it is recommended to conduct additional relevant analyses and to further specify the populations who should avoid or use with caution.

### Kidney disease progression

Given the effects of pharmacologic HIF activation in models of CKD appear to be context-dependent and less consistent, the concern that anemia therapy with roxadustat may worsen CKD in certain subgroups of patients was raised. A phase 3 trial demonstrated that roxadustat therapy was associated with a greater decline in kidney function compared with placebo. Accordingly, the annual rate of change in estimated glomerular filtration rate was −3.70 mL/min per 1.73 m2 with roxadustat, and −3.19 mL/min per 1.73 m2 with placebo (95% CI, −1.00 to −0.01; *P* = 0.046) (Fishbane et al., 2021). Conversely, a systematic review and meta-analysis enrolled 18 trials with a total of 8806 participants revealed that no significant difference was observed in the risk of kidney-related adverse events when comparing roxadustat with the placebo (RR 1.088; 95% CI, 0.980–1.209) or ESA (RR 0.968; 95% CI, 0.831–1.152), in DD- (RR 2.649; 95% CI, 0.201–34.981) or NDD- (RR 1.053; 95% CI 0.965–1.149) CKD patients (Raichoudhury and Spinowitz, 2021). Additionally, our study also demonstrated that roxadustat mitigated the progression of renal fibrosis by modulation of fibroblast growth factor 23 (Wang et al., 2025). These seemingly contradictory results can be reasonably explained. The renal effects of HIF-PHI treatment appear to be dose- and duration-dependent. Specifically, administration of high-dose HIF-PHIs or sustained HIF-1 activation was found to exacerbate renal injury (Li et al., 2019; Li et al., 2021). Thus, undergoing trials specifically evaluating the effect of roxadustat on kidney disease progression may be warranted.

### Malignancy risk

Despite the critical role of HIF-mediated hypoxia adaptation in tumor progression, roxadustat phase 3 studies have not shown any signals indicating cancer initiation and/or progression. In an open-label, phase 2 study evaluating the efficacy and safety of roxadustat in anemic patients receiving chemotherapy for non-myeloid malignancies, the results showed that roxadustat increased Hb without adverse events of tumor progression in patients with chemotherapy-induced anemia regardless of tumor type and chemotherapy regimen (Glaspy et al., 2023). Interestingly, the same results were achieved from a phase III randomized, open-label, controlled study evaluating the efficacy and safety of roxadustat for the treatment of chemotherapy-induced anemia in patients with non-myeloid malignancies (Lu et al., 2025). Furthermore, recently, the results of MATTERHORN study, a phase III, randomized, double-blind, placebo-controlled trial, preliminarily exploring the efficacy and safety of roxadustat for treating anemia in patients with lower risk-myelodysplastic syndromes, demonstrated that roxadustat was well tolerated (Mittelman et al., 2024). Collectively, there was a general view that no data so far do suggest any clinically relevant impact of roxadustat on malignancy risk. However, the accrued exposure time in clinical trials and clinical practice has not been long enough to be confident of the absence of a clinically relevant risk. Post-marketing surveillance will be critical to evaluate potential effect of roxadustat on cancer initiation and/or progression.

### Other safety concerns

As a key transcription factor for adaptive hypoxic responses, HIF also regulates numerous biological processes under physiological and pathological conditions. These include possible involvement in hyperkalemia, infections, proliferative retinopathy, pulmonary hypertension, low thyroid-stimulating hormone, vascular calcification, and cyst formation in polycystic kidney disease (Maxwell and Eckardt, 2016; Zhu et al., 2025). Notably, biochemical and crystallographic assays suggest that roxadustat has affinity to thyroid hormone receptor β and affects the negative feedback loop in the hypothalamic-pituitary-thyroid axis (Cheng et al., 2023; Yao et al., 2019). A retrospective cohort study in China reported cases of central hypothyroidism among patients receiving roxadustat treatment (Li et al., 2025). Therefore, it is recommended that in people with anemia and CKD treated with roxadustat, thyroid stimulating hormone and free thyroxine and triiodothyronine were monitored after 4 weeks of therapy initiation.

Collectively, these clinically significant adverse events associated with roxadustat may become more apparent as we gain experience with the use of roxadustat in clinical practice.

## Potential measures for mitigating safety risks

In clinical practice, roxadustat treatment for CKD patients with anemia requires targeted mitigation strategies to balance efficacy and safety risks. First, baseline assessment and periodic monitoring are critical. Accordingly, clinicians should assess Hb levels every 2–4 weeks during dose titration, avoiding Hb overshoot. Renal function, electrolyte panels and iron metabolism must also be tracked to adjust doses for patients with progressive CKD or iron deficiency. Second, patient stratification minimizes high-risk exposure. For instance, roxadustat should be used cautiously in individuals with a history of cardiovascular disease, or thrombosis. Third, in the initial treatment phase, therapy should be initiated at the minimum feasible effective dose to mitigate early adverse events. Additionally, drug-drug interaction screening is essential—concomitant administration of roxadustat with phosphate binders reduced exposure to roxadustat. Therefore, administrations should be separated by an interval of at least 1 hour. Finally, long-term post-marketing surveillance will clarify other potential risks (e.g., malignancy), ensuring ongoing refinement of safety protocols for roxadustat use in diverse CKD populations.

## Prospection and conclusion

Given the pleiotropic functions of HIF activation, pharmacologic activation of HIF in patients with anemia of CKD is also likely to have effects beyond erythropoiesis and iron metabolism. Notably, to what extent non-erythropoietic signaling pathways are activated in patients receiving roxadustat is difficult to predict, and the advantages of roxadustat must therefore be balanced against potential risks. More importantly, this study provides a theoretical framework and practical evidence for the clinical application of other HIF-PHIs.

In conclusion, pushed by the possibility that ESAs with a different mechanism of action might have a better safety profile, and to possibly improve the treatment of patients who responded poorly to ESAs, a new category of drugs was developed, HIF-PHIs. To date, it is generally recommended that roxadustat is superior to placebo and/or non-inferior to ESAs in correcting and/or maintaining hemoglobin concentration at target levels among patients with non-dialysis dependent CKD and incident and prevalent dialysis dependent CKD. Additionally, there are consensus that roxadustat is used in practice with no major signals of potential harm. Clinically significant advantages and adverse events may become more discernible as real-world experience with the use of roxadustat in clinical practice.

## Search strategy and selection criteria

To identify relevant literature for this review, we conducted a comprehensive search using Web of Science and PubMed databases. The search strategy employed combinations of keywords including “roxadustat”, “anemia”, “chronic kidney disease”, “hypoxia-inducible factor”, “HIF-PHI”, “safety”, “renal anemia”, “randomized controlled trial” and “inflammation”. The search was restricted to peer-reviewed original research articles and review papers published from 2012 to 2025. This review is not intended as a systematic review, but rather as a narrative synthesis informed by a targeted literature search and expert appraisal. The final reference list was curated based on relevance to the review’s objectives and selected through author consensus, rather than via systematic screening protocols.
